# LBX2-AS1 up-regulated by NFIC boosts cell proliferation, migration and invasion in gastric cancer through targeting miR-491-5p/ZNF703

**DOI:** 10.1186/s12935-020-01207-w

**Published:** 2020-04-26

**Authors:** Gang Xu, Yan Zhang, Na Li, Yanling Wu, Jinbiao Zhang, Rui Xu, Hui Ming

**Affiliations:** Oncology Department, The 960th Hospital of the PLA, No. 20 Zhanbei Road, Zibo, 255300 Shandong China

**Keywords:** LBX2-AS1, miR-491-5p, ZNF703, Gastric cancer

## Abstract

**Background:**

The crucial role of long non-coding RNAs (lncRNAs) has been certified in human cancers. The lncRNAs with abnormal expressions could act as tumor inhibitors or oncogenes in the advancement of tumors. LBX2-AS1 was once reported to accelerate esophageal squamous cell carcinoma. Nonetheless, its function in gastric cancer (GC) remained a riddle.

**Methods:**

RT-qPCR was used to examine the expression of NFIC/LBX2-AS1/miR-491-5p/ZNF703 in GC cell lines. The functions of LBX2-AS1 in GC were appraised by colony formation, EdU, flow cytometry analysis, transwell and wound healing assays. Luciferase reporter, ChIP and RNA pull down assays were utilized to evaluate the interactions among genes.

**Results:**

LBX2-AS1 was up-regulated in GC cell lines. Knockdown of LBX2-AS1 repressed the proliferative, migratory, and invasive abilities of GC cells. Moreover, LBX2-AS1 was transcriptionally activated by NFIC. And LBX2-AS1 could bind with miR-491-5p. Besides, miR-491-5p depletion or ZNF703 upregulation could counteract the repressing effects of LBX2-AS1 silence on GC progression.

**Conclusion:**

In a word, LBX2-AS1 up-regulated by NFIC promoted GC progression via targeting miR-491-5p/ZNF703, implying LBX2-AS1 was an underlying treatment target for GC patients.

## Background

Gastric cancer (GC) ranks in the third responding the mortality related to cancer, posing a tremendous threat to global human health [1]. With the laparoscopy of endoscope, the efficiency of surgical treatment obtained a huge improvement [2]. Although substantive achievements were made in the treatment, the morbidity had been keeping rising by years. Meanwhile, the prognosis of GC patients was not satisfactory. The primary reason was attributed to lack of early diagnosis [3]. Consequently, it was necessary to identify a biomarker for the early diagnosis of GC not only to improve accurateness of diagnosis but also to find a target for treatment.

Lately, the unique functions of long non-coding RNAs (lncRNAs) are discovered in multiple cancers [4, 5]. LncRNAs are a group of noncoding transcripts whose length is over 200 nucleotides, with limited capacities in coding proteins [6]. Accumulating evidence suggested that lncRNAs had played vital roles in a broad scale of biological movements among cancers, including apoptosis, metastasis and proliferation as well as chemoresistance [7–9]. Thus, it was exceedingly important to comprehend the pathology of GC by delving into potential mechanism of lncRNAs. LBX2-AS1 is a novel lncRNA and considered to exert oncogenic function in esophageal squamous cell carcinoma by promoting migration and epithelial-mesenchymal transition (EMT). However, the biological function of LBX2-AS1 has not been explored ever in GC.

Competing endogenous RNAs (ceRNA) network attracted more and more attention due to its significant effects on regulating the progression of cancers and non-tumor diseases [10, 11]. For example, HOTAIR served as a ceRNA to modulate HER2 expression via sponging miR-331-3p in GC [12]. ZEB1-AS1 repressed the process of GC via ceRNA network. TINCR modulated growth of GC via sponging miR-375 to up-regulate the expression of PDK1 [13]. This study planned to investigate whether LBX2-AS1 played a role of ceRNA in GC.

Hence, the current study focused on how LBX2-AS1 exerted functions in GC by regulating the downstream targets.

## Methods

### Cell lines

Human GC cell lines (MGC803, BGC823, HGC27 and SGC7901) and gastric epithelial cell line (GES1) were both procured from ATCC (Rockville, Maryland) and cultivated in the DMEM (Invitrogen, Carlsbad, CA). Cell culture was conducted with 1% Pen/Strep solution (Invitrogen) and 10% FBS (Gibco, Grand Island, NY) at 37 °C in 5% CO_2_. The culture medium was changed every 3 days.

### Total RNA isolation and real-time quantitative polymerase chain reaction (RT-qPCR)

Total RNAs from MGC803 and BGC823 cells were isolated in line with the protocol of TRIzol reagent (Thermo Fisher Scientific, Waltham, MA) for reverse transcription. SYBR Green PCR Master Mix (Takara, Kyoto, Japan) was then utilized for qPCR. Results were processed by 2^−ΔΔCT^ method and normalized to GAPDH or U6. Primers used here were: LBX2-AS1: Forward: 5′-CGTGGGGAATGGACCCATAG-3′, Reverse: 5′-GGACTTGCCCTTGGTGACTC-3′; miR-491-5p: Forward: 5′-AGTGGGGAACCCTTCCAT-3′, Reverse: 5′-CTCTACAGCTATATTGCCAGCCAC-3′; NFIC: Forward: 5′-TGGCGGCGATTACTACACTTCG-3′, Reverse: 5′-GGCTGTTGAATGGTGACTTGTCC-3′; ZNF703: Forward: 5′-TGCAGCCGCTGTCCTCCACTC-3′, Reverse: 5′-CACCGAGTTGAGTTTGGAGGAG-3′; GAPDH: Forward: 5′-ACCTGACCTGCCGTCTAGAA-3′, Reverse: 5′-GTCAAAGGTGGAGGAGTGGG-3′; U6: Forward: 5′-CTCGCTTCGGCAGCACA-3′, Reverse: 5′-AACGCTTCACGAATTTGCGT-3′.

### Transfection

MGC803 and BGC823 cells were collected for 48 h of transfection as per the guidebook of Lipofectamine 2000 (Invitrogen). The shRNAs specific to LBX2-AS1, pcDNA3.1/NFIC, miR-491-5p mimics, miR-491-5p inhibitor, pcDNA3.1/LBX2-AS1, pcDNA3.1/ZNF703 and their relative negative control (NC) sh-NC, pcDNA3.1, NC mimics and NC inhibitor, all these were produced by Genepharma (Shanghai, China). In detail, 2 μg of shRNAs (sh-NC, sh-LBX2-AS1#1/2) or pcDNA3.1 plasmids (pcDNA3.1 vector, pcDNA3.1/NFIC, pcDNA3.1/LBX2-AS1 or pcDNA3.1/ZNF703) were added into each well of a six-well plate. Additionally, cells were transfected with 50 nM mimics (NC mimics or miR-491-5p mimics) or 100 nM inhibitors (NC inhibitor or miR-491-5p inhibitor), respectively.

### Colony formation assay

MGC803 and BGC823 cells were incubated in the 6-well plates for 14 days, then fixed and dyed with 4% paraformaldehyde and 0.5% crystal violet (both for 15 min), severally. Colonies were counted manually.

### EdU incorporation assay

4 × 10^4^ GC cells were seeded in the 96-well plates with the ultra-low attachment surface and round bottom (Corning Inc., Corning, NY) for treating with EdU assay kit (Ribobio). After 4 h of incubation with 25 μM EdU medium diluent, cells were fixed via 4% paraformaldehyde for 30 min followed by DAPI staining for another 30 min. Thereafter, cells were pictured by fluorescence microscopy (Olympus, Tokyo, Japan).

### Flow cytometry analysis for apoptosis

Flow cytometry analysis for apoptosis was conducted in the MGC803 and BGC823 cells using Annexin V-fluorescein isothiocyanate (FITC)/PI double staining method. In brief, cells were treated with Annexin-V-FITC and propidium iodide (PI) for 15 min in succession. Apoptotic cells were analyzed with FACS Calibur (BD Bioscience, San Jose, CA).

### Western blot

Cell protein extracts were quantitated for treatment with electrophoresis on 10% SDS-PAGE and PVDF membranes, then with 5% nonfat milk. Membranes were then mixed with the primary antibodies (Abcam, Cambridge, MA) against Bcl-2 (ab32124, 1:1000 dilution), Bax (ab32503, 1:2000 dilution), Total caspase-3 (ab13847, 1:500 dilution), Cleaved caspase-3 (ab32042, 1:500 dilution), ZNF703 (ab188031, 1:1000 dilution) and control GAPDH (ab181602, 1:10,000 dilution) all night at 4 °C, following incubation with corresponding secondary antibodies tagged with HRP (ab20272, 1:2000 dilution) for 2 h. Protein band was examined by enhanced chemiluminescence (ECL) detection system (Bio-Rad, Hercules, CA).

### Transwell assay

GC cells were plated into the upper chamber of transwell inserts (Corning) coating with Matrigel or not for invasion or migration assay. Complete medium was added to the lower chamber. After incubation for 24 h, cells penetrating to the bottom were fixed and stained with crystal violet solution.

### Wound healing assay

Cells were cultured in 6-well plates (5 × 10^5^ cells/well) to 90% confluence. Scratches were later made through drawing two parallel lines using a 10 µl sterile pipette tip. Any cellular debris was obliterated through washing the cells thrice with PBS (Sigma-Aldrich, St. Louis, MO, USA). Migration area was detected at 0 and 24 h via a light microscope (Nikon, Tokyo, China).

### Dual-luciferase reporter assay

LBX2-AS1 promoter covering the wild-type (WT) or mutated (Mut) NFIC binding sites were cloned into the pGL3-Basic vector (Promega, Madison, WI), then co-transfected with pcDNA3.1/NFIC or pcDNA3.1 for 48 h. Besides, the pmirGLO reporter vector (Promega) containing the WT and Mut miR-491-5p binding sites within LBX2-AS1 or ZNF703 fragment were severally generated for luciferase assays. Finally, the dual-luciferase reporter assay system (Promega) was applied for detection of luciferase intensity.

### Chromatin immunoprecipitation (ChIP) assay

Cells of MGC803 and BGC823 treated with formaldehyde, then DNA–protein cross-links were sonicated into fragments of 200-1000 bp and received immunoprecipitate with NFIC-specific antibody and control anti-IgG (Millipore, Bedford, MA) overnight. The precipitated chromatin DNA was retrieved by adding beads and analyzed with RT-qPCR.

### Fluorescence in situ hybridization (FISH) assay

The fixed GC cells were rinsed in PBS and mixed with the LBX2-AS1 FISH probe (Ribobio) in the hybridization buffer overnight. Hoechst solution was added for nuclear counterstaining. Cells were measured with fluorescence microscopy.

### RNA pull down assay

Pierce Magnetic RNA–Protein Pull-Down Kit (Thermo Fisher Scientific, Waltham, MA) was applied for RNA pull down assay in GC cells following the recommendations of supplier. The WT or Mut miR-491-5p sequences containing LBX2-AS1 binding sites were synthesized and biotinylated to Bio-miR-491-5p-WT/Mut for incubation with cell protein samples overnight. The pull-down mixture was collected by beads for detecting the relative enrichment of LBX2-AS1 using RT-qPCR.

### RNA immunoprecipitation (RIP) assay

RIP assay was performed in line with the user manual of EZ-Magna RIP RNA Binding Protein Immunoprecipitation Kit (Millipore) using the specific antibodies against Ago2 and IgG as control. For immunoprecipitation, cell lysates were incubated with anti-Ago2 or IgG for one night. The protein samples being digested, precipitated RNAs were isolated and purified for RT-qPCR analysis.

### In vivo assay

3-week-old nude mice were acquired from the National Laboratory Animal Center (Beijing, China). They were subcutaneously injected with MGC803 cells with or without LBX2-AS1 silence. Animal research was conducted upon the approval of the Institutional Animal Care and Use Committee of the 960^th^ Hospital of the PLA. Tumor growth was analyzed by recording tumor volume every fourth day, and 28 days later, tumor volume and weight were examined after mice were sacrificed.

### Hematoxylin and Eosin (H&E) staining assay

The collected tissues from xenograft model were fixed with 4% paraformaldehyde at 4 °C, then embedded in paraffin and cut into 4 µm sections. After de-paraffin, sections were rehydrated and stained with H&E (Sigma-Aldrich) at 4 °C for 10 min, finally observed under Olympus light microscope.

### Immunohistochemistry (IHC) staining assay

Fresh tissues obtained from in vivo assay were fixed in paraformaldehyde. And then they were dehydrated in ethanol solutions, inset in paraffin and cut into 4-μm thickness. Afterwards, they were cultured with primary antibodies against Ki67 overnight at 4 °C, and then were cultivated with HRP-conjugated secondary antibodies. Finally, all these sections were visualized under microscope (Olympus).

### Statistical analysis

Continuous variables of 3 or more biological repeats were displayed as the mean ± SD. PRISM 6 (GraphPad, San Diego, CA) was employed to develop data analysis by Student’s t-test for two groups and by one-way/two-way ANOVA for more than two groups, with p-value less than 0.05 as significant level.

## Results

### LBX2-AS1 possessed a potent expression in GC cells and its knockdown restrained malignant phenotypes of GC cells

**Fig. 1:**
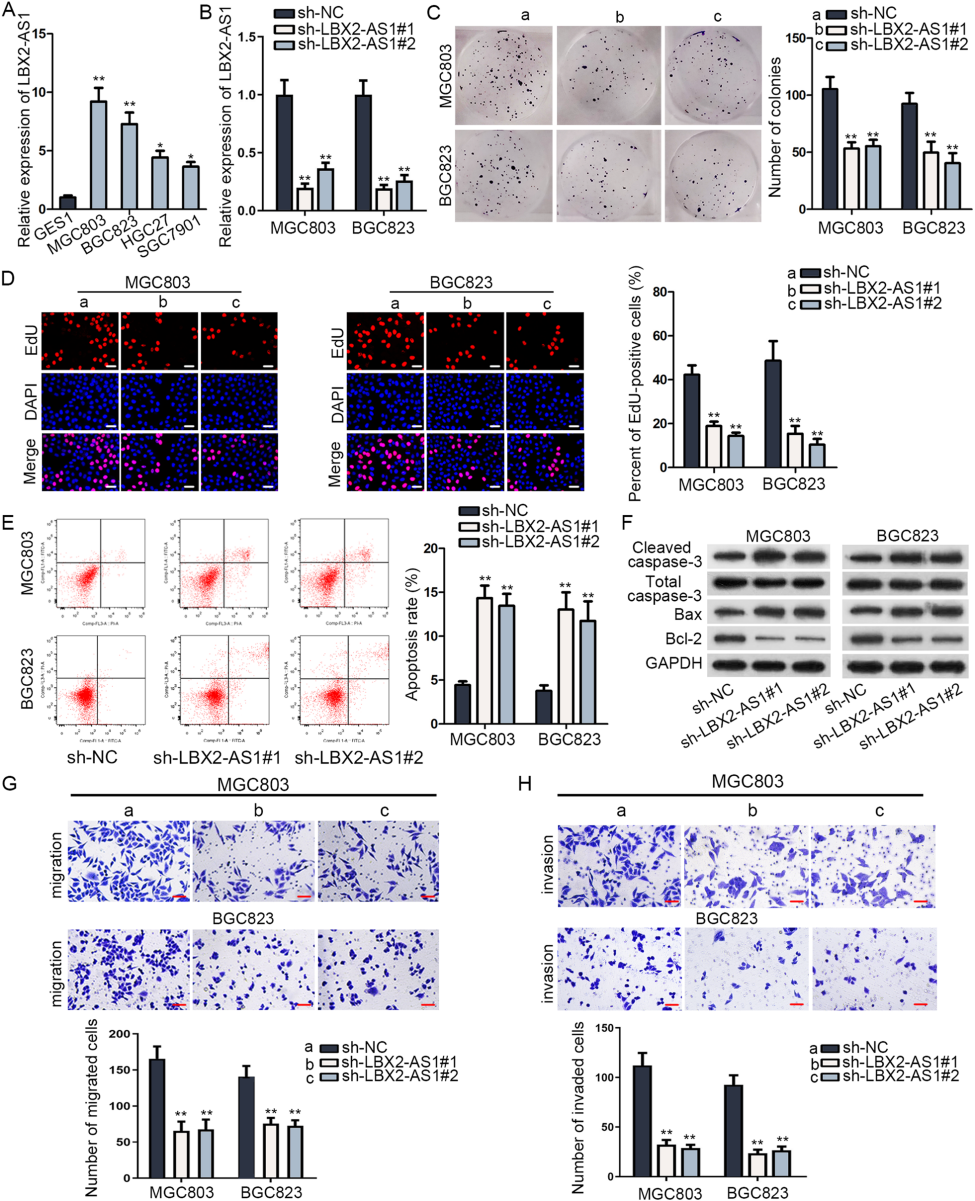
LBX2-AS1 possessed a potent expression in GC cells. **a** RT-qPCR analysis of LBX2-AS1 in GC cell lines and normal gastric cell. **b** Measurement of the efficacy of LBX2-AS1 knockdown via RT-qPCR in MGC803 and BGC823 cells. **c**, **d** Evaluation of proliferation ability of MGC803 and BGC823 cells transfected with sh-LBX2-AS1#1/2 or sh-NC via colony formation and EdU assays (scale bar = 200 μm). **e** Measurement of cell apoptosis capability in transfected cells via flow cytometry. **f** Evaluation of proteins associated with apoptosis by western blot in transfected cells. **g**, **h** Evaluation of cell migration and invasion capacities in transfected cells by transwell. Scale bar = 200 μm. ^*^P < 0.05, ^**^P < 0.01

To begin with, we uncovered the potential role of LBX2-AS1 in GC cells. We observed a differentially high expression of LBX2-AS1 in GC cell lines (MGC803, BGC823, HGC27 and SGC7901) via RT-qPCR (Fig. 1a). Since a more satisfactory efficiency of LBX2-AS1 knockdown was detected in sh-LBX2-AS1#1/2-transfected cells via RT-qPCR (Fig. 1b), we performed loss-of-function assays using sh-LBX2-AS1#1/2 to study the biological role of LBX2-AS1 in MGC803 and BGC823 cells. As illustrated in Fig. 1c, d, down-regulation of LBX2-AS1 repressed the proliferation of MGC803 and BGC823 cells. In addition, cell apoptosis rate was escalated by silencing LBX2-AS1 in MGC803 and BGC823 (Fig. 1e). Western blot was applied to measure the proteins correlated with apoptosis. Data revealed that cleaved caspase-3 and Bax protein levels were increased but Bcl-2 protein level was lessened by down-regulated LBX2-AS1 (Fig. 1f). More importantly, LBX2-AS1 silence weakened migratory and invasive capabilities of cells (Fig. 1g, h). Also, the wound healing assay presented similar results that inhibition of LBX2-AS1 dramatically blocked the migratory ability of GC cells (Additional file 1: Fig. S1A). More importantly, the growth rate of tumor in LBX2-AS1-silenced group was sharply diminished through in vivo experiments (Additional file 1: Fig. S1B-C). Furthermore, results of H&E staining and IHC staining assays revealed that metastatic nodules and Ki67-positive cells in LBX2-AS1-silenced group were decreased (Additional file 1: Fig. S1D). In a word, LBX2-AS1 was highly expressed in GC cells and knockdown of LBX2-AS1 suppressed GC cell growth and motility.

### NFIC activated LBX2-AS1 transcription in GC cells

**Fig. 2:**
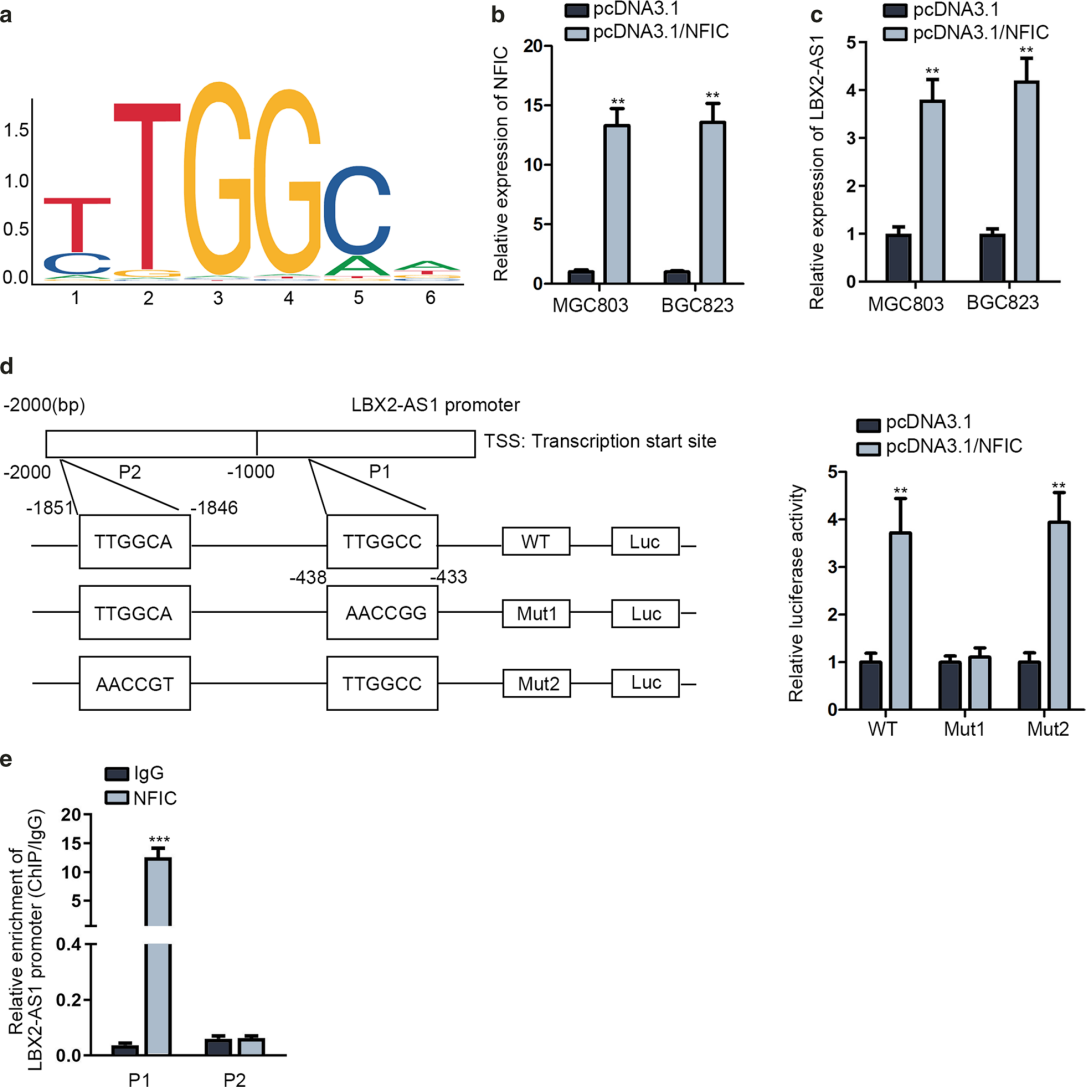
NFIC activated LBX2-AS1 transcription in MGC803 and BGC823. **a** The DNA motif of NFIC was exhibited. **b** The efficiency of NFIC overexpression via RT-qPCR assay. **c** Analysis of LBX2-AS1 expression in transfected cells via RT-qPCR. **d** The schematic diagram of the binding area of NFIC on LBX2-AS1 promoter (left). The interaction between LBX2-AS1 promoter and NFIC was verified through luciferase reporter assay (right). **e** ChIP assay assessed the enrichment of LBX2-AS1 promoter. ^**^P < 0.01, ^***^P < 0.001

After studying the crucial biological function of LBX2-AS1 on the progression of GC, we probed into the potentially molecular regulatory mechanism of LBX2-AS1 in GC. It was discovered in UCSC (http://genome.ucsc.edu/) that NFIC was a potential transcription factor of LBX2-AS1 (Additional file 2: Fig. S2). Among these factors, we focused on NFIC since it has been proved to regulate gene transcriptions previously [14, 15] and has also been reported to be related to the gastric tumorigenesis [16]. Afterwards, the DNA motif of NFIC and the binding site between LBX2-AS1 and NFIC were obtained from JASPAR (http://jaspar.genereg.net/) (Fig. 2a). Additionally, we observed that NFIC expression was up-regulated in response to pcDNA3.1/NFIC (Fig. 2b). To explore whether NFIC exerted a repressive or activating effect on LBX2-AS1 transcription, we utilized RT-qPCR at first and noted a significantly up-regulated expression of LBX2-AS1 in pcDNA3.1/NFIC transfected GC cells (Fig. 2c). Substantially, we divided LBX2-AS1 promoter into 2 pieces and marked them with P1 and P2. Data of luciferase reporter assays manifested that overexpression of NFIC could augment luciferase activity remarkably built with LBX2-AS1 promoter-WT. Next, we separately made P1 and P2 mutant partially and surprisingly found up-regulation of NFIC could increase the activity of LBX2-AS1 promoter Mut2 but not Mut1 (Fig. 2d). Therefore, P1 was confirmed as the binding area of NFIC on LBX2-AS1 promoter. Results of ChIP showcased that P1 was enriched in NFIC antibody but not in IgG while P2 depicted no evident changes in both (Fig. 2e). In summary, NFIC accelerated the LBX2-AS1 transcription in GC cells.

### LBX2-AS1 functioned as a ceRNA in GC cells

**Fig. 3:**
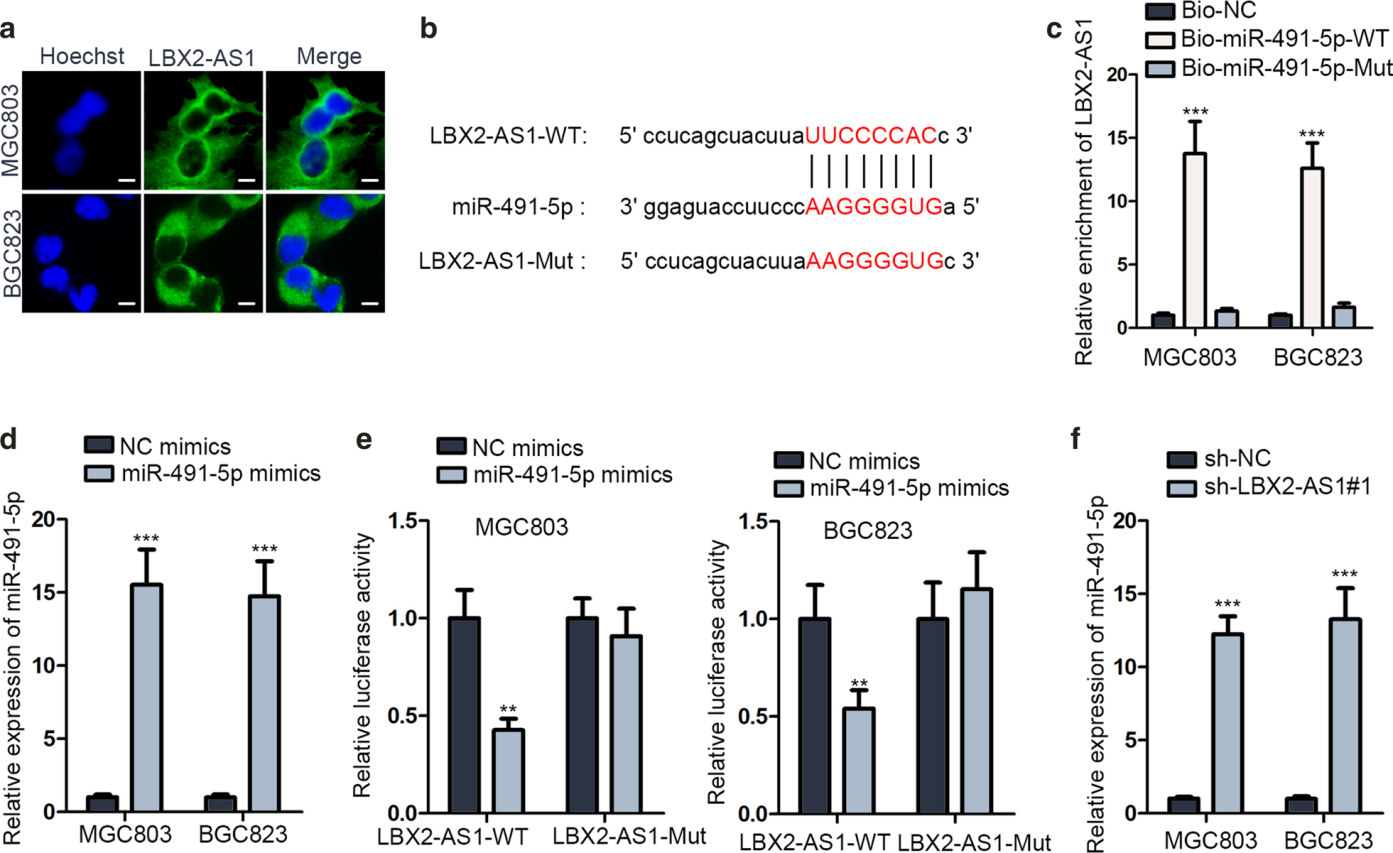
LBX2-AS1 functioned as a ceRNA in GC cells. **a** Cytoplasmic location of LBX2-AS1 in GC cells via FISH assay. Scale bar = 10 μm. **b** Bioinformatics presentation of the binding site between LBX2-AS1 and miR-491-5p. **c** RNA pull down examined the interaction between LBX2-AS1 and miR-491-5p. **d** MiR-491-5p overexpression efficiency was examined in MGC803 and BGC823 cells via RT-qPCR. **e** The interaction between LBX2-AS1 and miR-491-5p was verified by luciferase reporter assay. **f** RT-qPCR analysis of miR-491-5p expression in LBX2-AS1 silenced cells. ^**^P < 0.01, ^***^P < 0.001

To further investigate the underlying mechanism of LBX2-AS1 in GC, FISH assays were conducted and data disclosed that LBX2-AS1 was mainly distributed in cytoplasm of GC cells (Fig. 3a), suggesting that LBX2-AS1 possessed potential to post-transcriptionally modulate gene expression. According to the search results of starBase (http://starbase.sysu.edu.cn), total of 23 miRNAs was screened out as downstream candidates of LBX2-AS1 (Additional file 3: Table S1). Among those candidates, miR-491-5p has been revealed to suppress gastric carcinogenesis by previous researches [17, 18]. Therefore, we chose miR-491-5p to continue our study. Besides, the latent binding sites between miR-491-5p and LBX2-AS1 were shown in Fig. 3b. Results of RNA pull down exhibited that biotinylated miR-491-5p-WT could make LBX2-AS1 become sediment while biotinylated miR-491-5p-Mut failed (Fig. 3c). MiR-491-5p expression was seen to rise after transfecting miR-491-5p mimics (Fig. 3d). Outcomes of luciferase reporter assays displayed that miR-491-5p up-regulation could lessen the activity of LBX2-AS1-WT while no distinct changes were seen in that of LBX2-AS1-Mut (Fig. 3e). Moreover, the expression of miR-491-5p was hoisted in answer to down-regulation of LBX2-AS1 (Fig. 3f). Altogether, LBX2-AS1 was a ceRNA against miR-491-5p.

### LBX2-AS1 boosted process of GC via cutting down miR-491-5p

**Fig. 4:**
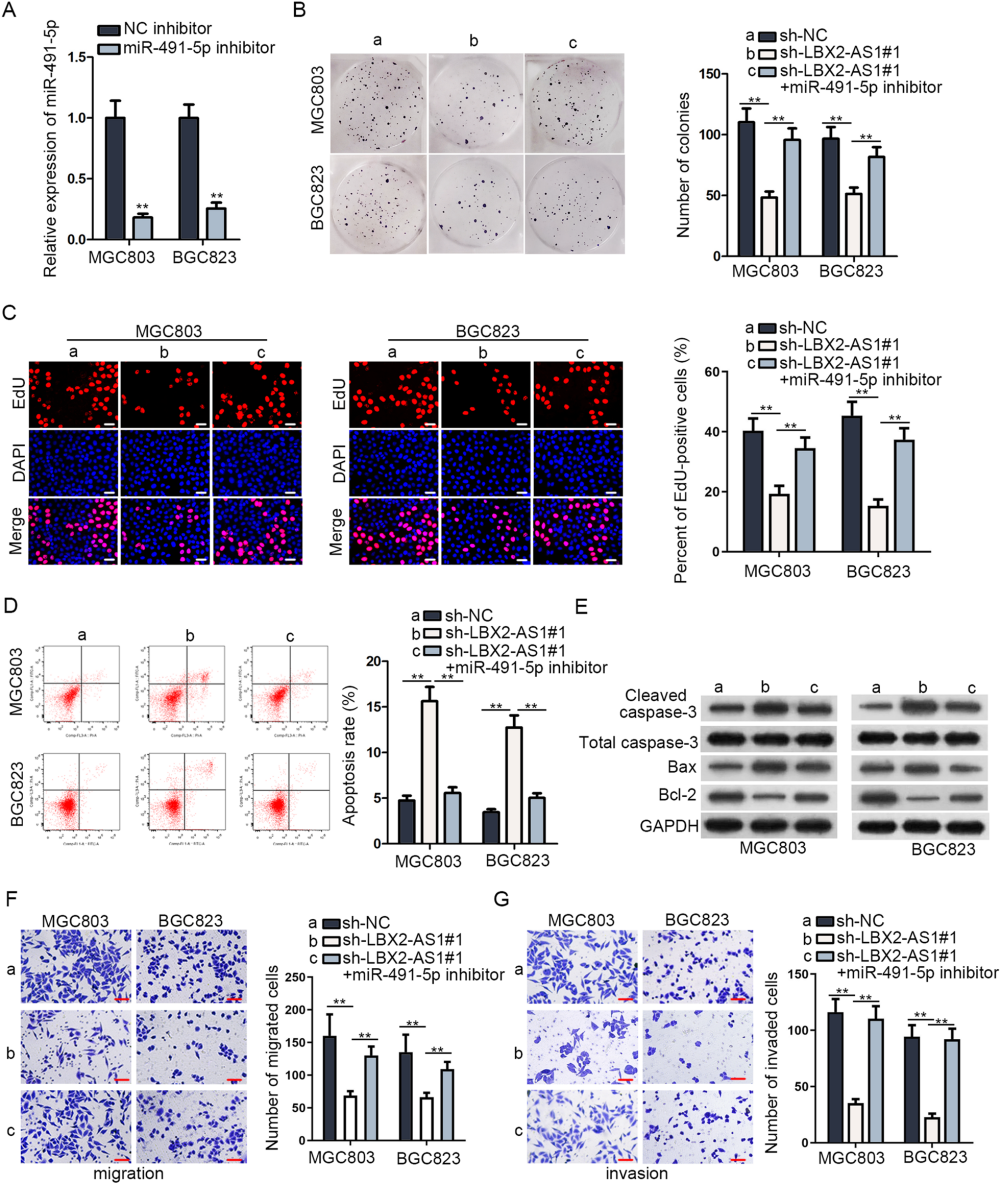
LBX2-AS1 boosted process of GC via cutting down miR-491-5p. **A** MiR-491-5p knockdown efficiency was examined by RT-qPCR in MGC803 and BGC823 cells. **B**, **C** Colony formation and EdU (scale bar = 100 μm) measured proliferation ability of GC cells treated with sh-NC, sh-LBX2-AS1#1, sh-LBX2-AS1#1 + miR-491-5p inhibitor. **D** Flow cytometry analyzed apoptosis rate in transfected cells. **E** Western blot measured expression of apoptosis-related proteins in transfected cells. **F**, **G** Transwell assays were built to test migration and invasion in transfected cells. Scale bar = 200 μm. ^**^P < 0.01

To confirm that LBX2-AS1 promoted GC via modulating miR-491-5p, at the beginning, RT-qPCR data showcased that miR-491-5p expression was dropped in reply to miR-491-5p inhibitor (Fig. 4A). As shown in Fig. 4B, C, miR-491-5p inhibitor restored the effects of LBX2-AS1 down-regulation on proliferation of MGC803 and BGC823 cells. Besides, the ascending apoptosis rate induced by LBX2-AS1 knockdown was counteracted by miR-491-5p down-regulation (Fig. 4D, E). Moreover, falling tendency of migratory and invasive capacities imposed by LBX2-AS1 silence were offset by miR-491-5p inhibitor (Fig. 4F, G). Besides, miR-491-5p inhibition offset the blocking effect of LBX2-AS1 depletion on cell migration in both MGC803 and BGC823 cells (Additional file 4: Fig. S3A). In brief, LBX2-AS1 fostered GC cell proliferation, migration and invasion through targeting miR-491-5p.

### LBX2-AS1 contributed to the course of GC via up-regulating ZNF703

**Fig. 5:**
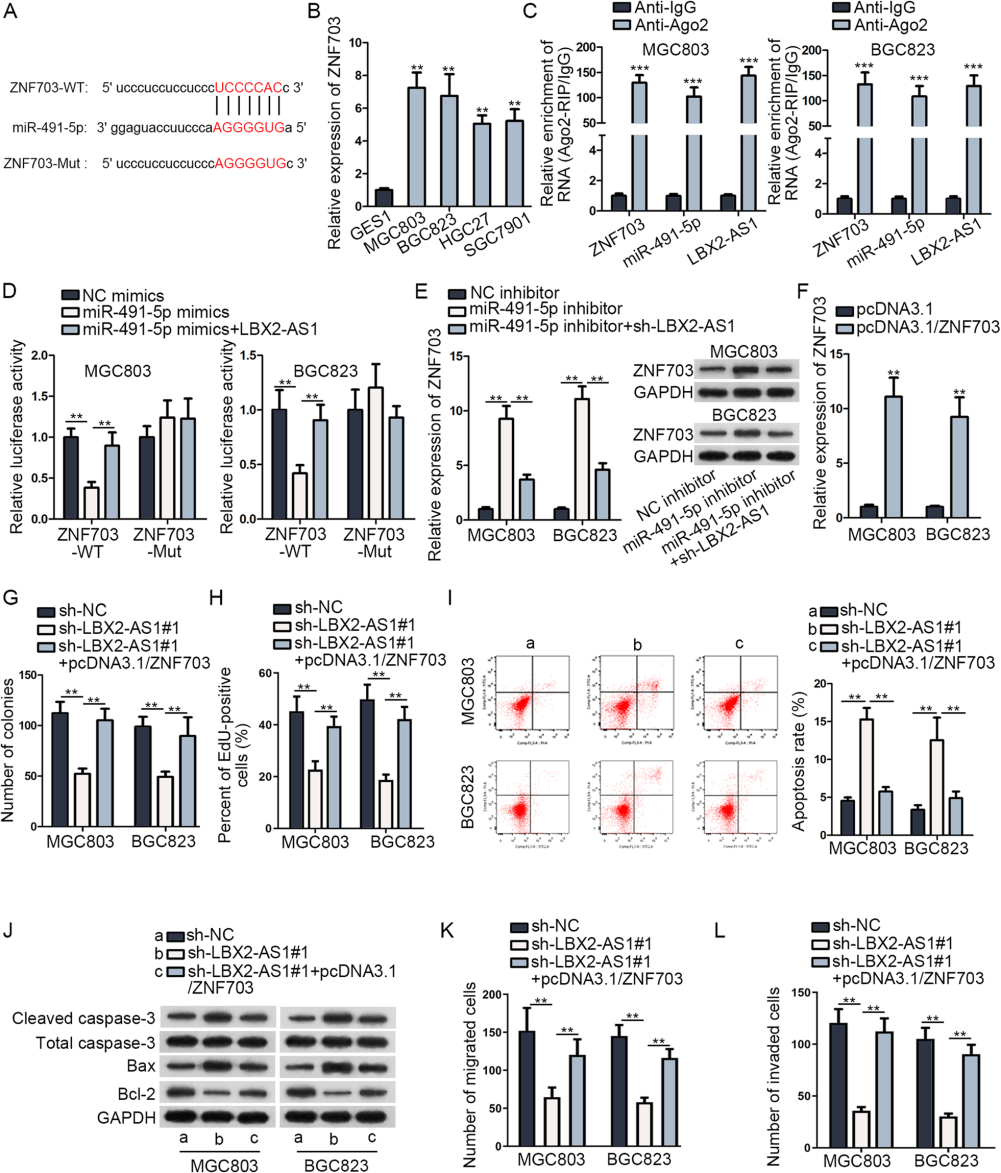
LBX2-AS1 contributed to the course of GC via up-regulating ZNF703. **A** Bioinformatics presentation of the binding site between miR-491-5p and ZNF703. **B** ZNF703 expression was evaluated by RT-qPCR in GC cell lines and normal gastric cell. **C** RIP assay testified the enrichment of LBX2-AS1, miR-491-5p and ZNF703 in anti-Ago2 or anti-IgG group. **D** Luciferase activity of ZNF703-WT/Mut in transfected cells was detected by luciferase reporter assay. **E** RT-qPCR and western blot were carried out to detect the mRNA and protein expressions of ZNF703 in transfected cells. **F** Overexpressed ZNF703 was validated in RT-qPCR. Colony formation (**G**), EdU (**H**), flow cytometry (**I**), western blot (**J**) and transwell assays (**K**, **L**) were performed to measure GC cell proliferation, apoptosis and migration as well as invasion in differently transfected groups. ^**^P < 0.01, ^***^P < 0.001

To explore molecules targeted by miR-491-5p, starBase tool was employed. In Additional file 5: Table S2, the potential targets of miR-491-5p were screened out (criterion: Degradome Data: high stringency; Program Number: 4 programs). Among these mRNAs, ZNF703 ranked first based on clipExpNum. Moreover, ZNF703 was reported to be an oncogene in GC cells [19]. Therefore, ZNF703 was chosen to as the target gene of miR-491-5p. Data from starBase (starbase.sysu.edu.cn) disclosed that ZNF703 had potential binding sites with miR-491-5p and the consequences were exhibited in Fig. 5A. We used RT-qPCR and observed that ZNF703 expression was up-regulated in GC cell lines (Fig. 5B). RIP assay results delineated that ZNF703, LBX2-AS1 and miR-491-5p got enrichment in Ago2 antibody but not in IgG antibody (Fig. 5C). After we overexpressed miR-491-5p in MGC803 and BGC823 cells, the luciferase activity of ZNF703-WT was diminished and then was recovered by LBX2-AS1 overexpression. Regarding the luciferase activity of ZNF703-Mut, no notable changes were observed among different groups (Fig. 5D). RT-qPCR and western blot were performed to detect the mRNA and protein expressions of ZNF703. Results disclosed that miR-491-5p down-regulation enhanced the mRNA and protein levels of ZNF703 while LBX2-AS1 silence reversed this rising trend (Fig. 5E). With the intention to verify that LBX2-AS1 exerted functions via ceRNA network by modulating ZNF703 in GC cells, we carried out rescue assays in MGC803 and BGC823. We used pcDNA3.1/ZNF703 to increase the expression of ZNF703 in MGC803 and BGC823 cells (Fig. 5F). Data of colony formation and EdU assays showed that the suppressed proliferative abilities caused by down-regulation of LBX2-AS1 could be countervailed by overexpression of ZNF703 (Fig. 5G, H and Additional file 4: Fig. S3B, C). Furthermore, up-regulation of ZNF703 could neutralize the promoting influence of LBX2-AS1 silence on apoptosis (Fig. 5I, J), migration and invasion (Fig. 5K, l and Additional file 4: Fig. S3D–F). Overall, LBX2-AS1 fostered the course of GC through enhancing the expression of ZNF703.

## Discussion

A huge body of essays have verified that lncRNAs exerted crucial imparts in the advancement of multiple cancers, including GC [20, 21]. For illustration, lncRNA HOXA11-AS promoted proliferation and invasion of GC cells by targeting miR-1297/EZH2 axis [22]. LncRNA DANCR facilitated motility via inhibition of lncRNAs-LET in GC cells [23]. LncRNA ZEB1-AS1 was reported to forecast unsatisfactory prognosis in GC [24]. These studies gave encouragement to discover the pathological process in GC. Moreover, LBX2-AS1 displayed unfavorable prognosis and facilitated proliferation and metastasis by Notch signaling in non-small cell lung cancer (NSCLC). Based on the previous researches, our study demonstrated that LBX2-AS1 was expressed in a high level in GC cells. Besides, LBX2-AS1 silence could hinder cell proliferation, migration as well as invasion in GC while promoted apoptosis. Considering regulatory mechanism underlying LBX2-AS1 in GC, we first discovered that cytoplasmic abundance of LBX2-AS1 in GC cells. Furthermore, we discovered that NFIC could stimulate the transcription of LBX2-AS1 in GC cells.

More importantly, lncRNAs are found to be involved in the carcinomas of various cancers through sponging special miRNAs and targeting mRNAs [25, 26]. For example, ZFAS1 contributed to GC progression through suppressing KLF2 and NKD2 expressions [27]. GACAT3 severed as a sponge of miR-497 to accelerate the course of GC [28]. Our study found that miR-491-5p was negatively regulated by LBX2-AS1 in GC and LBX2-AS1 sequestered miR-491-5p to facilitate the growth of GC.

Large quantities of works have certified that miRNAs are powerful regulators of their downstream mRNAs in cancers [29]. MiR-21 regulated prostaglandin signaling and accelerated GC via targeting 15-PGDH [30]. MiR-148b repressed glycolysis in GC by targeting SLC2A1 [31]. TEAD1/4 played an oncogenic role and was negatively modulated by miR-4269 in GC [32]. In this study, we found that miR-491-5p could negatively regulate the expression of ZNF703. MiR-491-5p constructed a bridge between LBX2-AS 1 and ZNF703.

ZNF703 was discovered to exert oncogenic function in numerous cancers, such as colorectal cancer [33], breast cancer [34] and cholangiocarcinoma [35]. In our study, we found that ZNF703 was positively modulated by LBX2-AS1 and ZNF703 up-regulation could rescue the effects of LBX2-AS1 silence on GC progression.

## Conclusion

In conclusion, it was validated in our study that NFIC-activated LBX2-AS1 accelerated proliferative, migratory and invasive abilities of GC cells by sponging miR-491-5p to up-regulate ZNF703, offering a biomarker for the improvement of GC treatment.

## Supplementary information

**Additional file 1: Figure S1** (A) Wound healing assays were performed to evaluate the impact of LBX2-AS1 silence on GC cell migration. Scale bar = 200 μm. (B) Representative images and corresponding growth curves of tumors. (C) Weight of tumors derived from mice injected with MGC803 cells with or without LBX2-AS1 inhibition. (D) H&E staining of metastatic nodules and IHC staining of Ki67 positive cells in above two groups. Scale bar = 200 μm. ^**^P < 0.01, ^***^P < 0.001.

**Additional file 2: Figure S2** Factors that might regulate the transcription of LBX2-AS1 were obtained from UCSC.

**Additional file 3: Table S1** List of LBX2-AS1-bond miRNAs predicted by starBase.

**Additional file 4: Figure S3** (A) The migratory ability of indicated cells was assessed via wound healing assays. (B-E) Corresponding original images of data in Fig. 5G, H, K, L was shown, respectively. Scale bar was 200 μm for images in Fig. S3C-E. (F) Wound healing assay carried out in transected MGC803 and BGC823 cells. ^**^P < 0.01.

**Additional file 5: Table** **2** List of miR-491-5p-targeted mRNAs predicted by starBase.

## Data Availability

Research data and material are not shared.

## Abbreviations

15-PGDH: 15-Hydroxyprostaglandin dehydrogenase
ANOVA: Analysis of variance
ceRNA: Competing endogenous RNAs
ChIP: Chromatin immunoprecipitation
DANCR: Differentiation antagonizing non-protein coding RNA
DMEM: Dulbecco’s modified eagle medium
ECL: Enhanced chemiluminescence
EMT: Epithelial–mesenchymal transition
EZH2: Enhancer of zeste 2 polycomb repressive complex 2 subunit
FISH: Fluorescence in situ hybridization
GACAT3: Gastric cancer-associated transcript 3
GC: Gastric cancer
H&E: Hematoxylin and Eosin
HOTAIR: HOX transcript antisense RNA
HOXA11-AS: Homeobox A11 antisense RNA
IHC: Immunohistochemistry
KLF2: Krüppel-like Factor 2
LBX2-AS1: Ladybird homeobox 2 antisense RNA 1
lncRNAs: Long non-coding RNAs
Mut: Mutated
NC: Negative control
NFIC: Nuclear factor I C
NKD2: Naked cuticle homolog 2
NSCLC: Non-small cell lung cancer
PDK1: Pyruvate dehydrogenase kinase 1
PVDF: Polyvinylidene fluoride
RIP: RNA immunoprecipitation
RISCs: RNA induced silencing complexes
RT-qPCR: Real-time quantitative polymerase chain reaction
SD: Standard deviation
SDS-PAGE: Sodium dodecyl sulfate polyacrylamide gel electrophoresis
SLC2A1: Solute carrier family 2 member 1
TEAD1/4: Transcriptional enhanced associate domain 1/4
TINCR: TINCR ubiquitin domain containing
WT: Wild-type
ZEB1-AS1: Zinc finger E-box binding homeobox 1 antisense RNA 1
ZFAS1: Zinc finger antisense 1
ZNF703: Zinc finger protein 703
