# Supplementary figures and images for: LBX2-AS1 up-regulated by NFIC boosts cell proliferation, migration and invasion in gastric cancer through targeting miR-491-5p/ZNF703
Source: Cancer Cell Int. 2020 Apr 26;20:136. doi: 10.1186/s12935-020-01207-w (PMC7183605; doi:10.1186/s12935-020-01207-w)

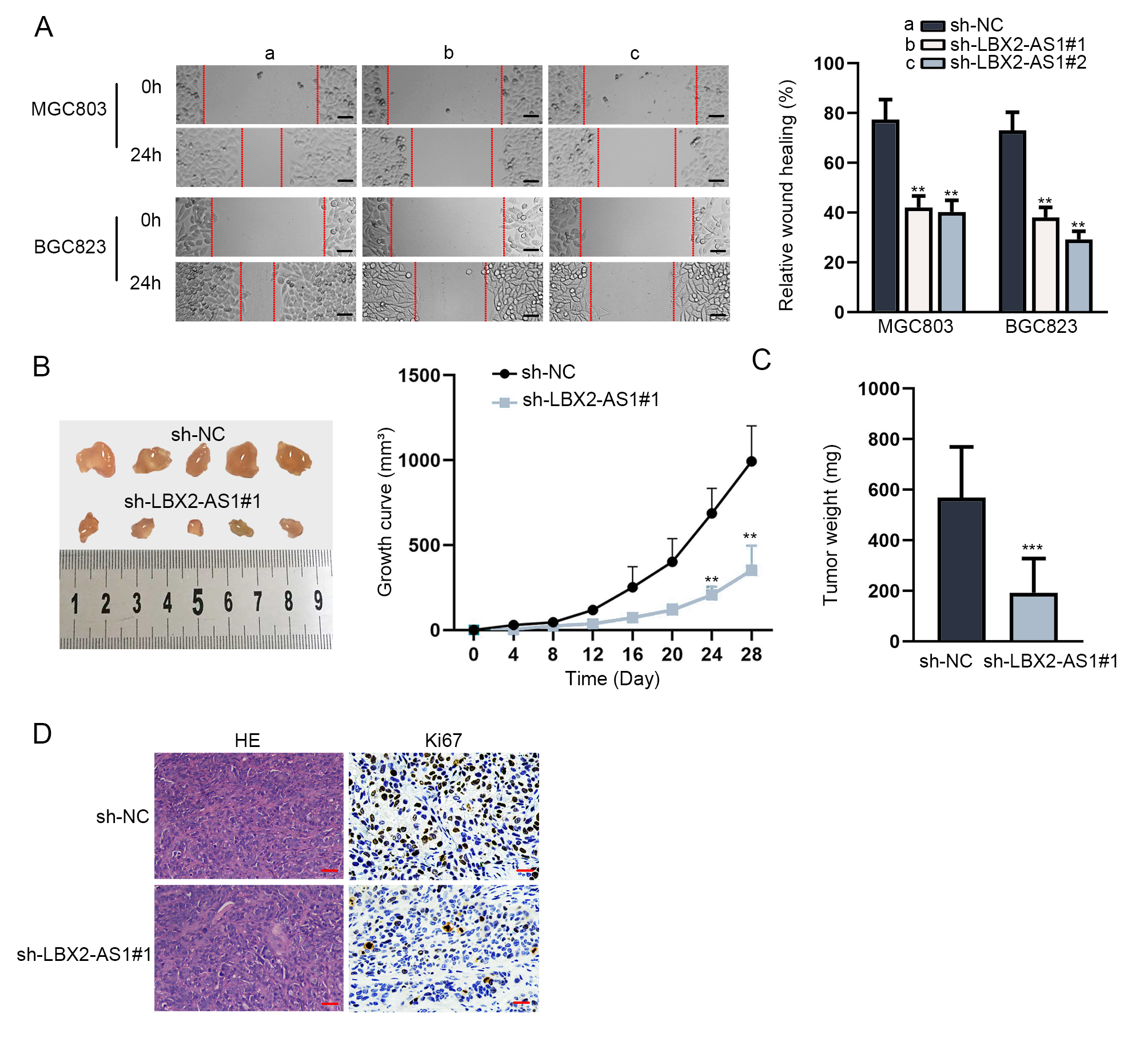

Supplement: Supplementary file 1 — Additional file 1: Figure S1 (A) Wound healing assays were performed to evaluate the impact of LBX2-AS1 silence on GC cell migration. Scale bar = 200 μm. (B) Representative images and corresponding growth curves of tumors. (C) Weight of tumors derived from mice injected with MGC803 cells with or without LBX2-AS1 inhibition. (D) H&E staining of metastatic nodules and IHC staining of Ki67 positive cells in above two groups. Scale bar = 200 μm. **P < 0.01, ***P < 0.001. [file 12935_2020_1207_MOESM1_ESM.tif]

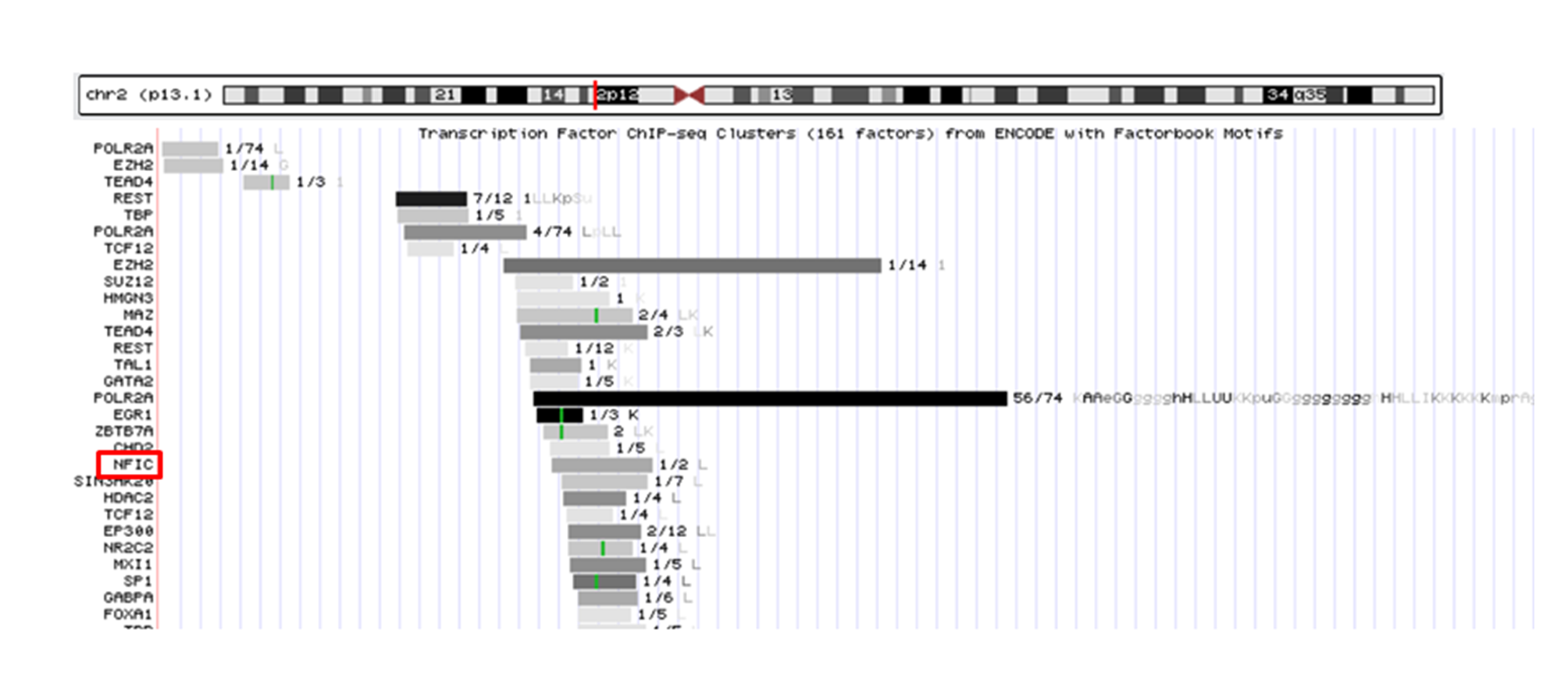

Supplement: Supplementary file 2 — Additional file 2: Figure S2 Factors that might regulate the transcription of LBX2-AS1 were obtained from UCSC. [file 12935_2020_1207_MOESM2_ESM.tif]

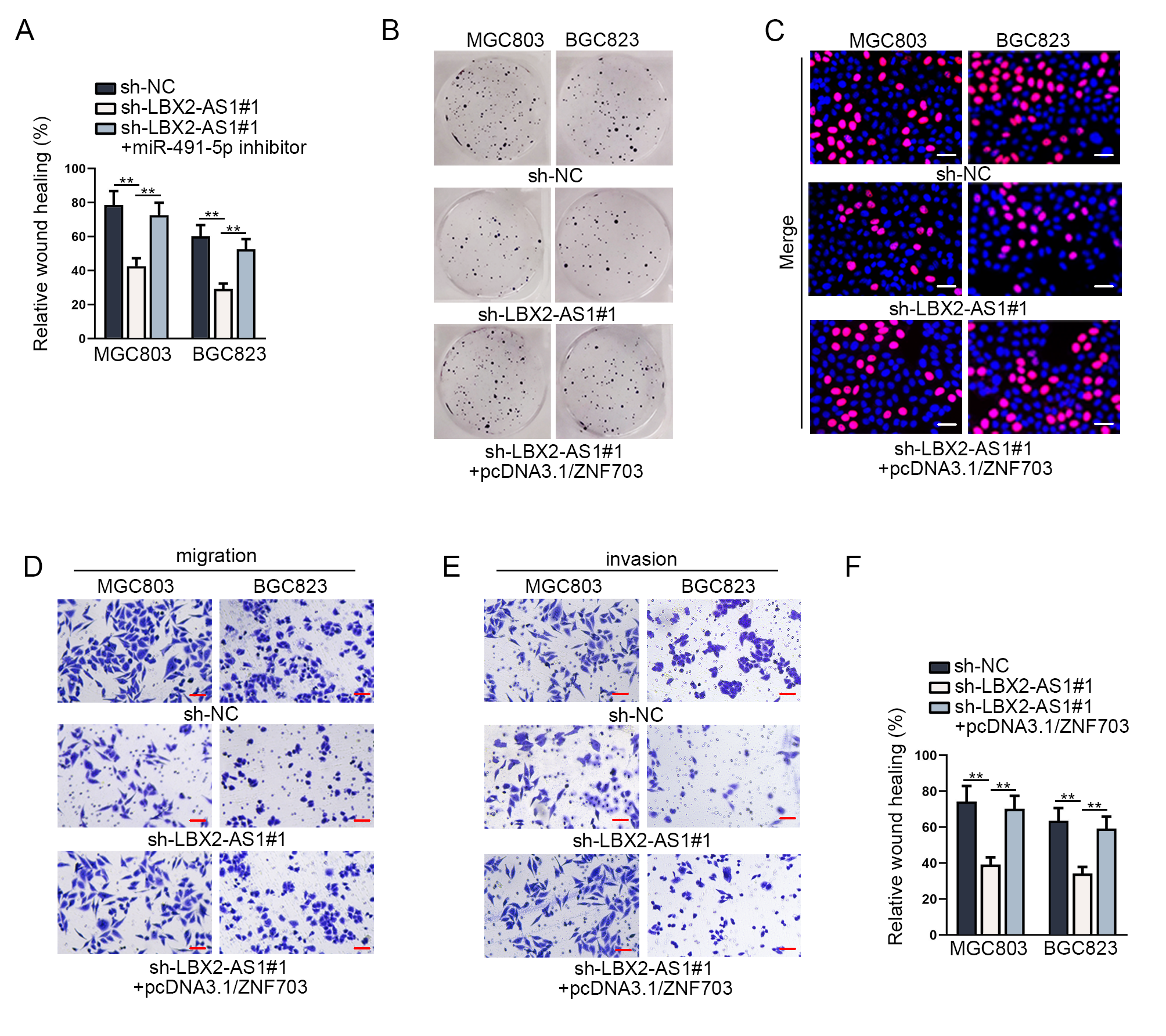

Supplement: Supplementary file 4 — Additional file 4: Figure S3 (A) The migratory ability of indicated cells was assessed via wound healing assays. (B-E) Corresponding original images of data in Fig. 5G, H, K, L was shown, respectively. Scale bar was 200 μm for images in Fig. S3C-E. (F) Wound healing assay carried out in transected MGC803 and BGC823 cells. **P < 0.01. [file 12935_2020_1207_MOESM4_ESM.tif]
